# Transcriptome–Metabolome Integration Deciphers the Metabolic and Transcriptional Reprogramming in Mice Due to *Vespa mandarinia* Venom

**DOI:** 10.3390/toxins18050198

**Published:** 2026-04-23

**Authors:** Jisu Jin, Guangyuan Jiao, Xiaolei Huang, Yingying Sun, Chao Chen, Hong Zhang

**Affiliations:** State Key Laboratory of Resource Insects, Institute of Apicultural Research, Chinese Academy of Agricultural Sciences, Beijing 100193, China; jinjisu9110@163.com (J.J.); jiaoguangyuan0206@163.com (G.J.); hlaym129@gmail.com (X.H.);

**Keywords:** *Vespa mandarinia*, transcriptomics, metabolomics, venom, BALB/c mice

## Abstract

Venom-mediated systemic toxicity is not fully understood. This study explored the dose-dependent effects of *Vespa mandarinia* venom (VMV) on mice via integrated transcriptomic and metabolomic analyses. Subcutaneous VMV injection induced dose-dependent hypothermia: 80 μg caused severe transient hypothermia and partial mortality, while 40/60 μg led to reversible hypothermia within 24 h. Whole-blood sequencing identified 2400–3281 differentially expressed genes (DEGs) per group, including 1764 shared DEGs. Immune-related pathways were significantly activated, with hub genes validated by qRT-PCR. Serum metabolomics revealed alterations in organic acids, alkaloids, and other metabolites. Integrative transcriptome–metabolome analysis predicted the potential involvement of various pathways in VMV-induced toxicity, including ferroptosis (shared in low-dose VMV groups) and apoptosis. Cumulatively, this study confirms that VMV induces immunometabolic reprogramming, providing a molecular framework for understanding venom-induced systemic toxicity.

## 1. Introduction

Hymenopteran venoms, including those from bees, wasps, and ants, are complex bioactive mixtures composed predominantly of peptide toxins, enzymes, and biogenic amines [1,2,3,4]. Envenomation most commonly causes immediate local inflammatory manifestations, including pain, erythema, edema, and pruritus. In the setting of multiple stings, high-dose exposure, or in susceptible individuals, venom can also elicit systemic toxic effects, such as nausea, hypotension, and acute kidney injury [5], and may trigger severe IgE-mediated allergic reactions [6]. Mechanistically, enzymatic components facilitate toxin dissemination through tissues, whereas membrane-active peptides can directly disrupt cellular membranes, leading to hemolysis and, in severe cases, tissue necrosis [7]. Clinical management primarily involves supportive care and targeted symptom relief. Mild reactions are generally managed with local cooling measures and oral antihistamines to alleviate pruritus and swelling [8,9]. If systemic anaphylaxis is suspected, prompt administration of intramuscular epinephrine is warranted [10]. For patients who meet established criteria, venom immunotherapy (VIT) should be considered to mitigate the risk of recurrent severe reactions after subsequent stings [11].

Research on hymenopteran venoms has recently shifted from single-component identification toward functional interrogation and integrative “omics” frameworks. Within this landscape, venomics has become key to understanding venom peptides, enzymes, toxin-like proteins, and their evolutionary relationships. For instance, modern omics methods such as mass spectrometry imaging have enabled rapid identification and spatial mapping of hymenopteran venom molecules [12]. Studies have also revealed the diverse proteins in parasitoid wasp venom and their evolutionary history [13]. Additionally, hymenopteran venoms are increasingly seen as sources of novel pharmacological leads and antimicrobial peptides. Research on the social wasp *Polybia paulista* venom identified enzymes with antioxidant properties and proteins related to immune response, inflammation, and allergy, including antigen 5, phospholipase A1, and hyaluronidase [14]. There is also growing interest in the “venom microbiome” and its ecological impact and potential medical applications [15]. Overall, the field is moving beyond compositional catalogs toward pharmacological function screening and downstream translational development.

While multi-omics studies are becoming more common, most research continues to focus on a single molecular level or specific organs, without integrating data across entire biological systems. For example, single-omics studies have explored gene expression changes after wasp (*Vespa basalis*) [16] or analyzed metabolic changes in blood after bee-venom exposure [17]. However, these studies do not connect the findings at multiple molecular levels. Meanwhile, Yuan et al. recently applied an integrative proteomics–metabolomics strategy to interrogate the pathophysiological mechanisms underlying wasp-sting-induced acute kidney injury (AKI) [18].

*Vespa mandarinia* venom (VMV) is a complex mixture of proteins, enzymes [4], low-molecular-weight bioactive peptides [19,20], and small molecules enriched in neuroactive amino acids [1,21]. Recent multi-omics studies of VMV have substantially advanced our understanding of the underlying toxicological mechanisms. Transcriptomic and metabolomic analyses, as well as their integrative interrogation, indicate that VMV exposure can provoke systemic disturbances, including impaired energy metabolism, oxidative stress, and aberrant activation of inflammatory signaling in animal models. For example, venom-gland transcriptome sequencing revealed high expression of toxin-associated genes, such as phospholipase A2 (PLA_2_), hyaluronidase, and neurotoxic peptides, providing a molecular basis for VMV pathogenicity [22]. In vivo, repeated multi-site subcutaneous administration of VMV induces AKI, accompanied by tubular damage and elevations in renal function markers [23]. Subsequent multi-omics profiling further supports that disruption of glutathione and purine metabolism constitutes a core mechanistic axis linking VMV exposure to rhabdomyolysis and nephrotoxicity [24]. Notably, pharmacological inhibition of PLA2 in VMV markedly attenuates metabolic dysregulation and oxidative injury, implicating PLA2 as a major toxicological target [25]. Mechanistic evidence indicates that STING signaling contributes to VMV-induced AKI by amplifying inflammatory responses and cellular injury [26]. Meanwhile, an α-helical peptide in VMV (VM peptide) exhibits antimicrobial activity and immunomodulatory effects in vivo, underscoring a dual profile spanning toxicity and therapeutic potential [27].

Collectively, multi-omics investigations suggest that VMV drives multi-organ injury through coordinated crosstalk among metabolic remodeling, oxidative stress, and inflammatory signaling, providing a conceptual basis for understanding systemic hymenopteran-venom toxicology and informing prevention and clinical management strategies. Despite these advances focused on a specific organ and select components, a critical gap remains: the systemic host response—particularly in BALB/c mice—under exposure to whole venom has not been comprehensively characterized in terms of coordinated metabolic network regulation and physiological readouts, such as body temperature, during acute toxic stress.

Building on these observations, we aimed to systematically delineate host defense and injury mechanisms elicited by VMV in BALB/c mice. Following a single subcutaneous injection of VMV, we monitored body temperature—an essential physiological readout of systemic allergy and metabolic shock—together with survival outcomes. We integrated transcriptomic and metabolomic datasets to construct a systems-level molecular regulatory network in response to VMV exposure. We further employed qRT–PCR to validate changes in the expression of key genes within representative pathways, such as inflammatory signaling and energy metabolism, identified by the multi-omics network analysis. Collectively, this research offers a comprehensive, systems-level perspective on the host’s molecular strategies for coping with the VMV challenge, thereby providing a rationale for the clinical management of *Vespa mandarinia* stings and identifying candidate therapeutic targets.

## 2. Results

### 2.1. The Effect of VMV Injection on Body Temperature Changes in Mice

**Figure 1:**
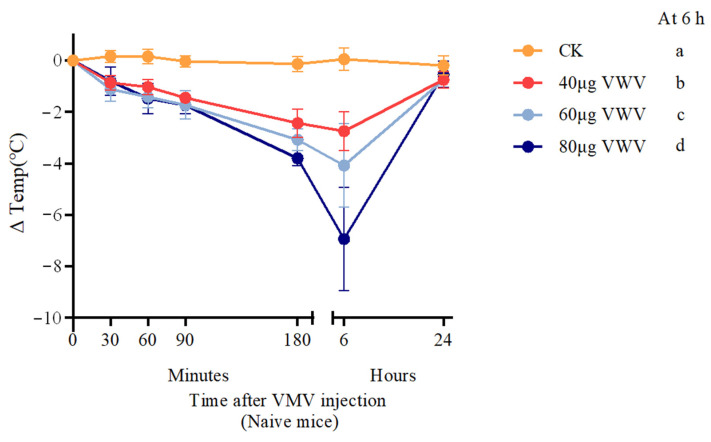
Effect of VMV injection on body temperature in mice. Female BALB/c mice were treated subcutaneously (s.c.) with the indicated doses of VMV, while control mice received PBS. Changes in body temperature (∆Temp, mean ± SEM) were monitored. Data were analyzed by two-way repeated-measures ANOVA, with post hoc multiple comparisons performed relative to the PBS-treated group. Different letters (a–d) indicate significant intergroup differences in ΔTemp at the 6 h time point (*p* < 0.05). Groups with distinct letters differ significantly. Linear regression analysis verified a significant dose-dependent linear trend between VMV dosage and the magnitude of hypothermia (R^2^ = 0.78, *p* < 0.001). Data are pooled from two independent experiments. Differences are considered statistically significant at *p* ≤ 0.05.

After administering different doses of VMV to naïve mice, we observed that their body temperature decreased with increasing injection dose during the first 6 h, reaching its lowest point at 6 h (Figure 1; 40 μg vs. 60 μg, *p* < 0.05; 60 μg vs. 80 μg, *p* < 0.01; 40 μg vs. 80 μg, *p* < 0.001). The control group (200 μL phosphate-buffered saline (PBS)) showed no significant change in body temperature (ΔTemp ≈ 0 °C) over the experimental period, confirming that the vehicle itself had no effect on thermoregulation.

The VMV 80 group exhibited the most profound hypothermic effect, reaching a nadir of approximately −7.0 °C at 6 h post injection (*p* < 0.01), followed by partial recovery by 24 h. The VMV 60 and VMV 40 groups showed intermediate and milder hypothermic responses, with ΔTemp values of approximately −4.0 °C and −3.0 °C at 6 h, respectively (*p* < 0.001). Hypothermia in the VMV 60 and VMV 40 treatment groups was transient, with body temperatures returning to baseline levels within 24 h.

### 2.2. Identification of DEGs and DEMs

**Figure 2:**
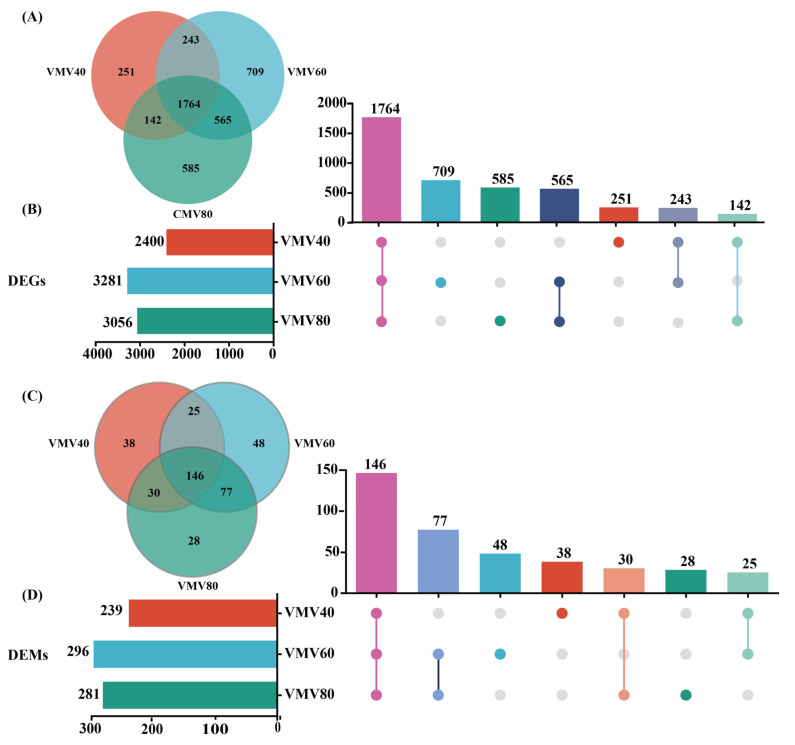
Venn diagram and UpSet plot of DEGs and DEMs. (**A**) Shared and unique DEGs among VMV and PBS treatment groups in peripheral blood (whole blood; FDR < 0.05). (**B**) Shared and unique DEGs among VMV and PBS treatment groups in peripheral blood (whole blood; FDR < 0.05). (**C**) Shared and unique DEMs among VMV and PBS treatment groups in peripheral blood (whole blood; *p* < 0.05). (**D**) Shared and unique DEMs among VMV and PBS treatment groups in peripheral blood (whole blood; *p* < 0.05). Horizontal bars indicate the number of DEGs or DEMs per VMV injection and the size of intersection sets are indicated using vertical bars. A single dot and its associated horizontal bar indicate the amount of genes or metabolites that are specific to a given dataset and have no commonality with other datasets used in the comparisons. DEGs, gene differential expression; DEMs, differently expressed metabolites.

Principal component analysis (PCA) revealed a clear distinction between the three treatments (VMV 40 vs. PBS, VMV 60 vs. PBS, VMV 80 vs. PBS) in peripheral blood (whole blood) samples. All raw data from the samples passed quality control (QC; Figures S2 and S3). A total of 2400 (1748 upregulated and 652 downregulated), 3281 (1954 upregulated and 1327 downregulated), and 3056 (1875 upregulated and 1181 downregulated) differentially expressed genes (DEGs; false discovery rate [FDR] < 0.05 & |log2(FC)| > 1) were identified. Comparison across the VMV 40, VMV 60, and VMV 80 groups in peripheral blood revealed 1764 significant overlapping DEGs (FDR < 0.05; Figure 2A,B).

**Figure 3:**
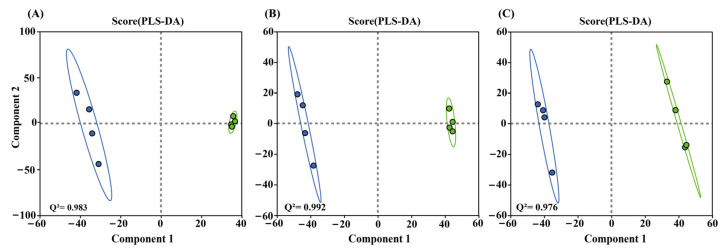
Two-dimensional scatter score plot and permutation test results for PLS-DA. (**A**–**C**) PLS-DA score plots for VMV 40 vs. PBS, VMV 60 vs. PBS, and VMV 80 vs. PBS (positive/negative ion mode), with the corresponding R^2^Y and Q^2^ values labeled in each plot.

Both positive and negative ion mode differences between the serum of the two treatment groups (VMV or PBS) were significant according to partial least squares discriminant analysis (PLS-DA) (Figure 3A–C). The PLS-DA models showed high explanatory and predictive ability with R2Y and Q2 values of VMV 40 vs. PBS (positive and negative ion modes) of (0.999, 0.983), VMV 60 vs. PBS of (1, 0.992), and VMV 80 vs. PBS of (0.999, 0.976). All Q^2^ values exceeded 0.5, indicating reliable and stable models. Validation via 200 permutation tests confirmed that all regression intercepts for Q^2^ were below 0.05 (Figure S4), indicating no evidence of overfitting.

Using a threshold of PLS-DA VIP > 1 and *p* < 0.05, 239, 296, and 54 differentially expressed metabolites (DEMs) were identified in serum samples for the VMV 40, VMV 60, and VMV 80 groups, respectively. Additionally, a comparison among these groups found 146 significant overlapping DEMs (*p* < 0.05) in both ion modes (Figure 2C,D).

### 2.3. KEGG and GO Functional Enrichment Analyses of DEGs

**Figure 4:**
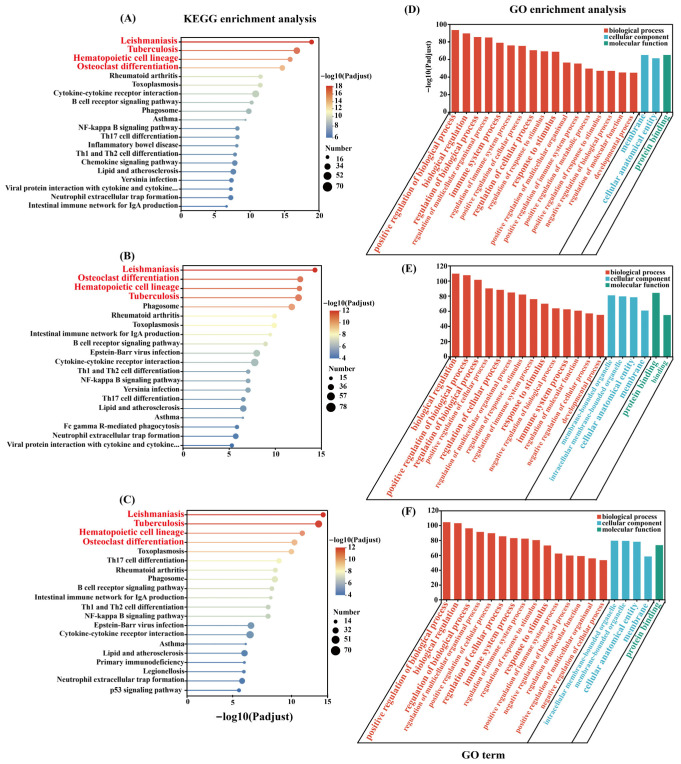
KEGG pathway (**A**–**C**) and GO functional enrichment (**D**–**F**) analyses of DEGs (top 20). (**A**,**D**) VMV 40 vs. PBS; (**B**,**E**) VMV 60 vs. PBS; (**C**,**F**) VMV 80 vs. PBS. For KEGG plots: Each point represents a pathway; ordinate = pathway name; abscissa = −log10(P_adjust) (smaller P_adjust = more significant enrichment); point size = number of DEGs in the pathway; color = range of −log10(P_adjust). For GO plots: Ordinate = −log10(P_adjust) (enrichment significance); abscissa = GO terms; colors represent biological process (BP), cellular component (CC), and molecular function (MF). Statistical significance: P_adjust < 0.05.

As shown by Figure 4A–C, the Kyoto Encyclopedia of Genes and Genomes (KEGG) functional enrichment analysis of DEGs with varying doses of injections showed the enriched pathways in the top 20. The further analysis revealed that the Leishmaniasis signaling pathway (mmu05140), Tuberculosis signaling pathway (mmu05152), hematopoietic cell lineage signaling pathway (mmu04640) and osteoclast differentiation signaling pathway (mmu04380) were significantly enriched across all treatments (Figure 4A–C).

The functional analysis with Gene Ontology (GO) showed that some GO terms were shared between the three treatment groups (VMV 40 vs. PBS, VMV 60 vs. PBS and VMV 80 vs. PBS). Common GO terms included biological processes (BP) such as positive regulation of a biological process (GO:0048518), biological regulation (GO:0065007), regulation of a biological process (GO:0050789), regulation of cellular process (GO:0050794), immune system process (GO:0002376), and response to stimulus (GO:0050896); molecular functions (MF) such as protein binding (GO:0005515); and cellular components (CC) such as membrane (GO:0016020) and cellular anatomical entity (GO:0005575) (Figure 4D–F).

### 2.4. Protein Interaction Network Analysis

**Figure 5:**
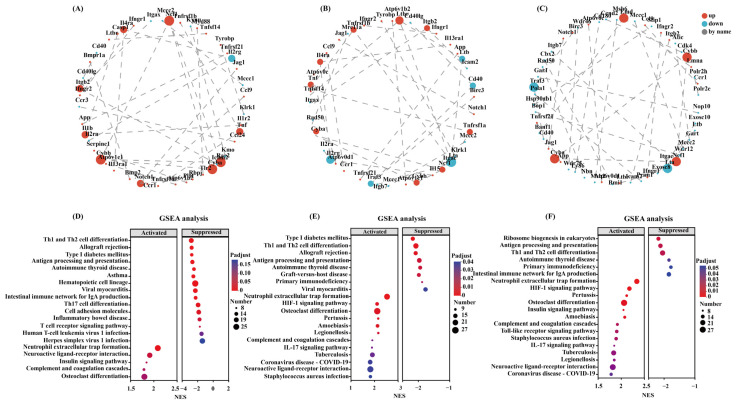
Protein–protein interaction (PPI) network of DEGs (top 30) and Gene Set Enrichment Analysis (GSEA) of DEGs (top 20). PPI networks for (**A**) VMV 40 vs. PBS, (**B**) VMV 60 vs. PBS, and (**C**) VMV 80 vs. PBS (*p* adj < 0.05, |log2(fold change)| > 1). Each node represents a gene, and edges represent interactions between genes. Node size is proportional to degree, with larger nodes indicating higher connectivity and greater potential importance within the network. Bubble plots of GSEA results for (**D**) VMV 40 vs. PBS, (**E**) VMV 60 vs. PBS, (**F**) VMV 80 vs. PBS. The abscissa represents the normalized enrichment score (NES), and the ordinate indicates gene set names. Point size reflects the number of genes within each gene set, and color corresponds to different P_adjust ranges. “Activated” denotes gene sets with NES > 0, whereas “suppressed” denotes gene sets with NES < 0.

The protein–protein interaction (PPI) networks were created based on an online STRING database (http://string-db.org/, accessed on 12 March 2025) and visualized in Cytoscape (https://cytoscape.org/; Cytoscape software version 3.7.2). The key genes were selected based on the CytoHubba and all the molecular nodes and edges were determined. Also, to plot and discover the genes of interest, the NetworkX library in Python has been used. The multiscale curvature classification algorithm has been used to identify the top 30 key genes. The top 30 key genes were obtained using the multiscale curvature classification algorithm. The results showed that the interactions among the top 30 DEGs across the three VMV treatment groups, as well as the 16 significantly overlapping DEGs, were explored using PPI analysis (Figure 5A–C).

### 2.5. Gene Set Enrichment Analysis (GSEA) of DEGs

Gene Set Enrichment Analysis (GSEA, http://software.broadinstitute.org/gsea, accessed on 20 March 2025) ranked the DEGs by differential expression of DEGs in the three samples and determined whether predefined gene sets were over-represented at the beginning or the end of the ranked list. Th1 and Th2 cell differentiation, antigen processing and presentation, and neutrophil extracellular trap formation were significantly enriched in all three VMV treatment groups. Cluster analysis of all DEGs in these pathways is depicted in grouped bubble charts (Figure 5D–F).

### 2.6. Validation of Gene Expression of the Differentially Expressed mRNAs by qRT-PCR

In order to verify whether the RNA sequencing results were accurate or not, the chosen five mRNA transcripts were randomly picked out of 18 highly overlapping DEGs (*p* < 0.05), and the qRT-PCR analysis was conducted. Extraction of RNA in the mouse peripheral blood followed by synthesis of cDNA was carried out after treating with VMV or PBS in accordance with the protocol. The five mRNAs were quantified using TransStart Green qPCR SuperMix Kit with SYBR (Transgen Biotech) on QuantStudioTM1 ABI Real-Time PCR Machine (Applied Biosystems by Thermo Fisher Scientific, New York, NY, USA).

**Figure 6:**
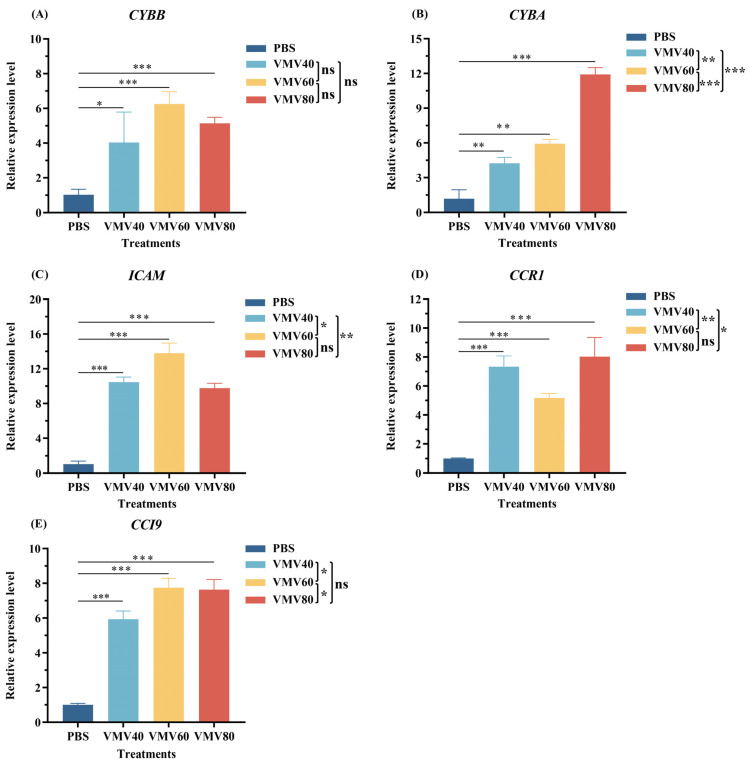
Validation of differentially expressed mRNAs by qPCR. The transcriptomic results for *Icam*, *Cybb*, *Cyba*, *Ccl9*, and *Ccr1* were confirmed by qPCR across the four treatments (VMV vs. PBS; *n* = 3 per group). (**A**) Expression level of *Cybb*, (**B**) the expression level of *Cyba*, (**C**) the expression level of *Icam*, (**D**) the expression level of *Ccr1*, and (**E**) the expression level of *Ccl9*. Data are presented as 2^−∆∆Ct^ values (mean ± SD), and the data were analyzed using Student’s *t*-test. * *p* < 0.05, ** *p* < 0.01, *** *p* < 0.001, and ns, not significant.

The mRNA levels of *Icam2*, *Cybb*, *Cyba*, *Ccl9*, and *Ccr1* were significantly upregulated in peripheral blood (*p* < 0.001); the expression trends were consistent with the RNA-seq data (Figure 6).

### 2.7. Metabolomics Analysis of Different Dosages of VMV Injections

**Figure 7:**
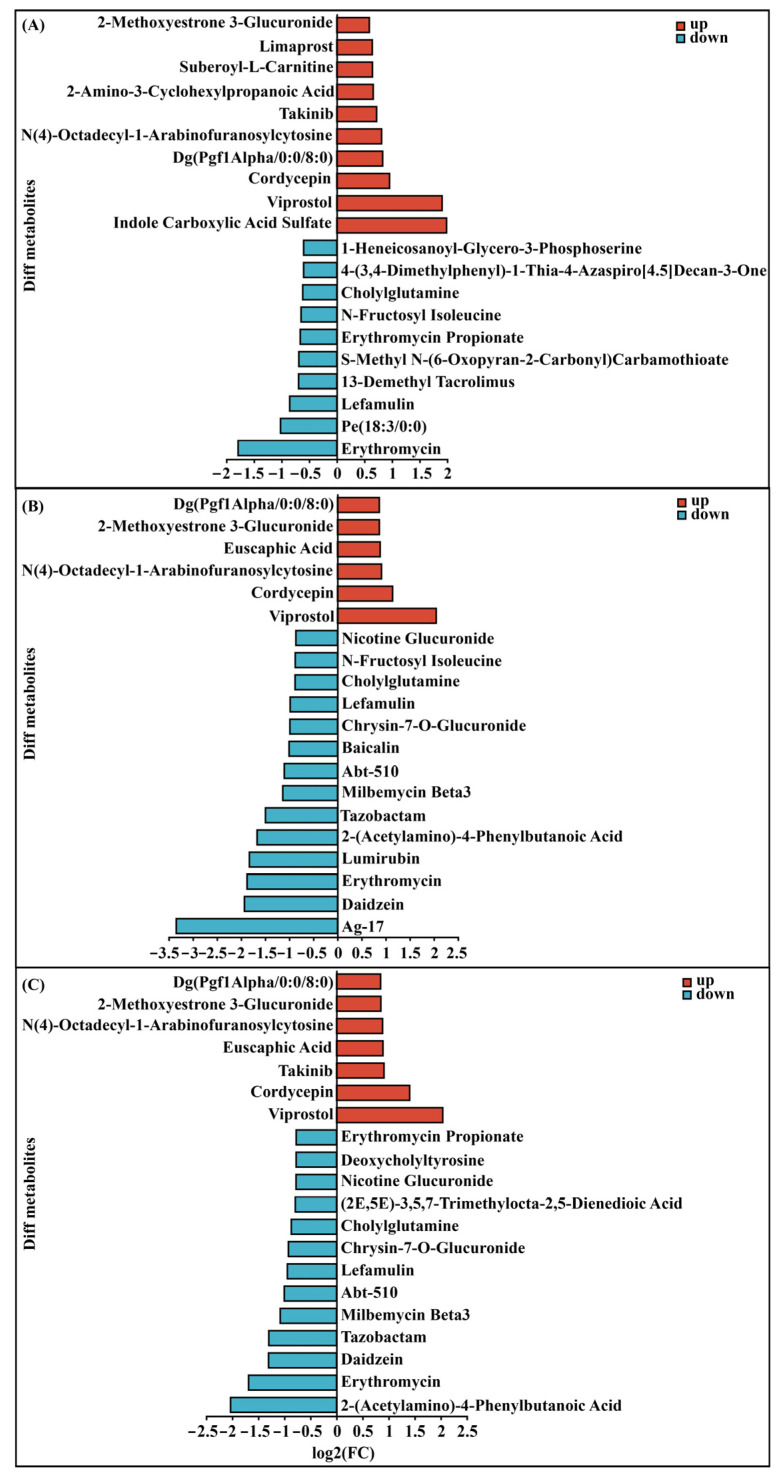
Fold-change analysis of DEMs (top 20). Plots for (**A**) VMV 40 vs. PBS, (**B**) VMV 60 vs. PBS, (**C**) VMV 80 vs. PBS. DEMs are examined using OPLS-DA, with variable importance in projection (VIP > 1) and Student’s *t*-test (*p* < 0.05) used as screening criteria. The abscissa represents log_2_ FC, and the ordinate displays metabolite names. Red indicates upregulated DEMs, and green indicates downregulated DEMs. Bar length corresponds to the magnitude of the fold-change.

Histogram analysis of the DEMs showed significant changes in peripheral blood following VMV injections. Further analysis indicated that DEMs predominantly comprised amino acids and derivatives, lipids and lipid-like molecules, nucleosides and analogs, organic acids and derivatives, as well as putatively annotated compounds, including 2-amino-3-cyclohexylpropanoic acid, limaprost, N(4)-octadecyl-1-arabinofuranosylcytosine, and putatively annotated compounds viprostol, cordycepin, and erythromycin (Figure 7). Several metabolites were putatively annotated as erythromycin, viprostol, and cordycepin and showed significant alterations following VMV treatment. Specifically, the erythromycin-annotated signal likely represents a gut microbial metabolite, suggesting that VMV induced gut dysbiosis and systemic metabolic stress. The viprostol-like annotation is likely an endogenous eicosanoid, with its modulation indicating activation of inflammatory lipid mediator pathways. Similarly, the cordycepin-like signal likely reflects an endogenous nucleoside isomer whose alteration is associated with VMV-induced tissue injury and disturbances in purine metabolism.

### 2.8. Integrative Analysis of the Metabolome and Transcriptome

**Figure 8:**
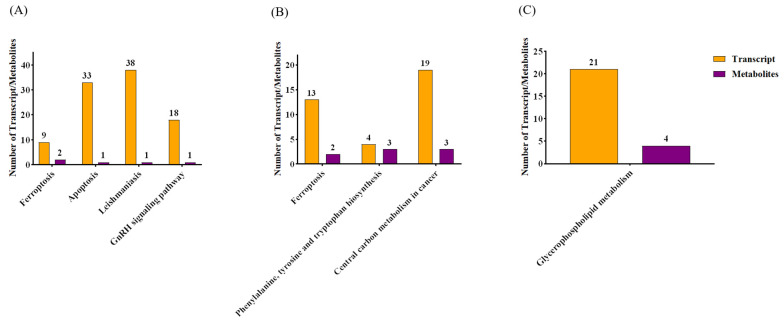
Histograms (**A**–**C**) of pathways co-enriched in DEGs and DEMs. (**A**) VMV 40 vs. PBS; (**B**) VMV 60 vs. PBS; (**C**) VMV 80 vs. PBS. Statistical criteria: DEGs (FDR < 0.05, |log2(FC)| > 1); DEMs (VIP > 1, *p* < 0.05).

All DEGs and DEMs with significant *p* adj < 0.05 and |log2(FC)| > 1, and OPLS-DA VIP > 1 and *p* value < 0.05, were considered. The analysis based on the Spearman correlation states that there is a high correlation between DEGs and DEMs (Figure S1). KEGG pathway enrichment analysis shows that coenriched pathways of DEGs and DEMs in the VMV40 group included ferroptosis, apoptosis, leishmaniasis, and GnRH signaling pathways; ferroptosis, phenylalanine, tyrosine, and tryptophan biosynthesis, and central carbon metabolism in cancer pathways were found in the VMV60 group whereas glycerophospholipid metabolism pathway was present in the VMV80 group (Figure 8A–C). Notably, ferroptosis was also among the co-enriched pathways between DEGs and DEMs in the low dose groups of VMV40 and VMV60. To conclude, four, three and one commonly shared KEGG pathways were identified between DEGs and DEMs in VMV40, VMV60 and VMV80 groups, respectively (Figure 8A–C).

## 3. Discussion

Our findings demonstrated that VMV induced a dose-dependent, transient hypothermic response in naïve mice, consistent with previous findings that venom can disrupt thermoregulation in mammals [28]. We observed a significant linear dose–response relationship between venom dosage and hypothermia severity—higher venom levels led to greater temperature reduction, likely due to synergistic effects among venom components. Bee venom is a complex mixture of peptides and enzymes, including melittin, PLA_2_, and mast cell degranulating peptides, each of which affects mammalian physiology in distinct ways [29]. Compounds similar to mastoparan and MCD peptides reportedly induce mast cell degranulation and histamine release, thereby affecting vascular and neuronal activity [29,30]. These immunomodulatory effects may contribute to altered heat generation or dissipation after venom exposure. Additionally, the partial recovery at 24 h suggests reversible physiological effects and venom clearance or inactivation, consistent with clinical observations that systemic symptoms of hymenopteran envenomation typically resolve within one to two days with supportive care [31]. This transient thermal dysregulation establishes a framework for further mechanistic studies and supports the use of this model in preclinical venom research.

By integrating transcriptomics and metabolomics, we used a comprehensive systems-biology approach to dissect the complex molecular mechanisms induced by exogenous interventions. Our multi-omics approach mapped systemic changes in peripheral blood after VMV injections at varying dosages (40, 60, and 80 μg). PCA and PLS-DA clearly distinguished VMV-treated groups from the PBS controls, demonstrating the reliability of our experimental model [32]. At the transcriptional level, we identified thousands of DEGs—ranging from 2400 to 3281 across treatment groups—suggesting that VMV triggered widespread reprogramming of gene expression in peripheral blood cells. Notably, 1764 DEGs overlapped across all three dosage groups, indicating a consistent transcriptional signature regardless of VMV dosage. Such conserved transcriptional signatures are more reliable indicators of the host’s fundamental response to VMV than those unique to specific doses, offering valuable targets for further investigation into VMV-induced toxicity [33].

Serum metabolomics revealed significant shifts in metabolic profiles, with core DEMs conserved across doses. Given the well-documented enzymatic activity of bee venom PLA_2_ in lipid metabolism, the observed metabolic shifts were likely driven, in part, by lipid metabolism remodeling—particularly phospholipid hydrolysis and changes in amino acid metabolism—though this hypothesis remains to be directly tested with pure bee venom PLA_2_ [34]. The concordance between extensive gene upregulation and distinct metabolic shifts highlights a tightly coordinated gene–metabolite interplay. Furthermore, the identification of overlapping signatures in both positive and negative ion modes reinforced the robustness of the metabolic impact. The ability of honey bee venom to alter serum metabolites suggests that local injection elicits systemic metabolic reprogramming, which may underlie its broad therapeutic effects in systemic diseases such as rheumatoid arthritis or neuroinflammation [33,35,36]. The finding that the intermediate dosage group exhibited the most extensive molecular changes warrants further investigation. This suggests that specific dosages may represent a pivotal threshold for inducing maximal systemic modulation, beyond which compensatory feedback mechanisms might be engaged to maintain homeostasis.

KEGG and GO enrichment analysis highlighted VMV’s role as a complex, multi-target systemic biological modulator. In this study, the Leishmaniasis (mmu05140) and Tuberculosis (mmu05152) pathways were consistently enriched across all dosages, illustrating broad, non-disease-specific innate immune activation in mice. Although these pathways are classified as pathogen-associated in the KEGG database, their presence does not indicate a disease-specific response in the current study, as the mice were not exposed to the corresponding pathogens. Instead, both pathways share a core gene set responsible for pattern recognition receptor (PRR) activity, pro-inflammatory cytokine secretion, and myeloid cell immune activation—key functional components of innate immunity. Their enrichment indicates that VMV strongly triggers universal innate immune and inflammatory signaling.

This aligns with the known pharmacological effects of honey bee venom’s (BV) main components, melittin and PLA2, which activate toll-like receptor signaling and modulate the NF-κB inflammatory cascade [37]. Additionally, the hematopoietic cell lineage (mmu04640) and osteoclast differentiation (mmu04380) pathways were significantly enriched, indicating VMV’s substantial impact on myeloid cell maturation and bone–immune crosstalk. Previous research has demonstrated that BV can inhibit RANKL-induced osteoclastogenesis by suppressing inflammatory signaling pathways, supporting its involvement in bone metabolism pathways [38].

GO functional analysis further corroborated these findings, showing shared BPs predominantly related to “immune system process” (GO:0002376) and “response to stimulus” (GO:0050896). The prominence of “biological regulation” and “regulation of cellular process” terms suggests that VMV triggers a structured regulatory response rather than uncontrolled inflammation. Enrichment for “membrane” (GO:0016020) and “protein binding” (GO:0005515) reflects VMV toxins’ robust interaction with cell surfaces and subsequent activation of intracellular signaling networks [32]. Collectively, VMV functions as a systemic “biological response modifier,” coordinating transcriptional programs that bridge innate immunity and metabolic homeostasis.

Metabolomic profiling revealed that VMV elicited substantial systemic alterations in mice, primarily influencing the concentrations of amino acids, lipids, nucleosides, and organic acids. The marked shifts in organic acid profiles suggested that VMV triggered metabolic stress and a transition in energy homeostasis, consistent with the known oxidative impact of Hymenoptera toxins [39,40]. The identification of putatively annotated compounds, such as cordycepin-like analogs and prostaglandin-related signatures (viprostol analogs), suggested modifications in nucleoside metabolism and inflammatory signaling cascades [41]. Considering erythromycin is a bacterial antibiotic, viprostol a synthetic prostaglandin analog, and cordycepin a characteristic fungal metabolite, these findings likely indicate a structurally similar endogenous or gut microbiota-derived isobaric compounds rather than exogenous exposure to the respective pharmaceutical or microbial agents. These systemic modifications were likely attributable to the combined action of venom PLA_2_ and hyaluronidases, which disrupted cellular membranes and facilitated the release of bioactive small molecules into circulation [42,43,44]. Collectively, these distinctive metabolic signatures provide a molecular basis for understanding the systemic toxicity and immunomodulatory potential of VMV.

This study has certain limitations that should be acknowledged. First, the interpretation of conserved transcriptional signatures relied solely on transcriptomic and metabolomic data without functional validation. Second, the potential involvement of the ferroptosis pathway was inferred exclusively from KEGG enrichment analysis of multi-omics data, lacking direct experimental assessment of classic ferroptosis indicators (e.g., lipid peroxidation, GPX4 expression, serum iron levels). Future studies will prioritize functional experiments to confirm core molecular mechanisms of VMV-induced toxicity, including verification of ferroptosis-related parameters to establish their definitive role.

In conclusion, this study provides a thorough investigation of differences in mRNA and metabolite levels across various VMV injection doses in a mouse model. Integrated metabolomics and transcriptomics provide detailed molecular insights into peripheral blood in this model. A comprehensive analysis of these molecular changes could help identify common pathways and elucidate the underlying mechanisms of the mouse response to subcutaneous VMV injection.

## 4. Materials and Methods

### 4.1. Mice

Six- to seven-week-old female BALB/c mice were procured from Beijing Vital River Laboratory Animal Technology Co., Ltd., located in Beijing, China. These mice were then accommodated at the Experimental Animal Center of the Beijing Institute of Animal Science and Veterinary Medicine, affiliated with the Chinese Academy of Agricultural Sciences. The housing conditions were carefully regulated, with a temperature maintained at 23 degrees Celsius plus or minus 2 degrees and a humidity level of 60% plus or minus 10%. Moreover, the mice had unrestricted access to both food and water.

### 4.2. Venom Preparation and Injection, and Temperature Detection

VMV was commercially obtained from Jianchang Nongxue Apiculture and Breeding Professional Cooperative (Huludao, China). The venom was collected via electro-stimulation, whereby a mild electrical stimulus was applied to hornets to elicit venom secretion; droplets were collected from the stingers. The crude venom samples were pooled, immediately frozen, and lyophilized for storage. Before each experiment, the lyophilized venom was reconstituted in sterile PBS to achieve the working concentrations (40, 60, or 80 μg per 200 μL). No additional purification steps were performed, as the objective of this study was to assess the toxicological properties of the whole venom complex.

After one week of acclimation, mice were subcutaneously (s.c.) injected with either 40 μg, 60 μg, or 80 μg of VMV in 200 μL of sterile PBS, or with 200 μL of PBS alone as a control (*n* = 6 per group). Body temperature was measured with a rectal thermometer immediately before and at various times after the injection.

The Ethics Committee of the Institute of Apicultural Research, Chinese Academy of Agricultural Sciences, approved all mice-related protocols (Approval No.: MFSLLSC-2026-002, dated 3 February 2026). Animal studies strictly adhered to China’s Laboratory Animal Welfare Guidelines (GB/T 35892-2018) [45].

### 4.3. Sample Collection and Processing

Injections were performed following the protocol in Section 4.2 (*n* = 4). At two time points (before injection and 6 h post injection), the mice were euthanized in accordance with animal welfare guidelines. Whole blood was promptly collected from the ophthalmic vein. In order to perform transcriptomic analysis, the 150 uL of whole blood was mixed with 750 uL of TRIzol and vigorously shaken and immediately frozen in liquid nitrogen (15 min) and kept at −80 °C. In order to conduct a metabolomic analysis, the whole blood was permitted to coagulate and divide at room temperature (37 °C) within an hour. The supernatant was obtained after centrifuging samples at 3000 rpm and 4 °C for 10 min and stored at −80 °C.

### 4.4. Transcriptome Examination and Analysis

All preparations of RNA were made using TRIzol reagent (Thermo Fisher Scientific, Waltham, MA, USA) as per the recommendations of the manufacturer with respect to the total RNA of whole blood. The purification, reverse transcription, library construction and sequencing were done at Shanghai Majorbio Bio-pharm Biotechnology Co., Ltd. (Shanghai, China) according to the protocol provided by the manufacturer (Illumina, San Diego, CA, USA). The DEGs were analyzed with DESeq2 and those whose FDR value was less than 0.05 and log2FC was equal to or greater than 1 were considered significantly DEGs. To conduct GO and KEGG analysis, respectively, these DEGs were analyzed with Goatools software (https://github.com/tanghaibao/GOatools, version: v1.2.3, accessed on 20 March 2025) and Python SciPy package (https://scipy.org/install/, version: 1.11.1, accessed on 20 March 2025). Fisher’s exact test with *p*-values of less than 0.05 was used to perform statistical analysis, indicating statistically significant enrichment of GO terms and KEGG pathways.

### 4.5. Non-Targeted Metabolomics Analysis

Processing of the 100 uL serum was performed using 300 uL methanol–acetonitrile solution (1:1, *v*/*v*). Sonication was performed at 40 kHz and five protein precipitation steps were carried out at 20 °C for 30 min each, followed by 30 min. To separate proteins in the supernatant, centrifugation was performed at 13,000× *g* and 4 °C. The supernatant was evaporated with nitrogen flow gradually and reconstituted with a loading solvent of acetonitrile–water (1:1, *v*/*v*) by sonication in a 5 °C water bath. Centrifugation was finally performed on the products at 13,000× *g* and 4 °C, after which LC-MS/MS was performed. All sample metabolites were of equal volume and pooled to prepare a QC sample.

LC-MS analysis was performed using a UHPLC-Exactive240 system equipped with an HSS T3 column. Chromatographic conditions were as follows: column: ACQUITY UPLC HSS T3 (100 mm × 2.1 mm i.d., 1.8 µm; Waters, Milford, MA, USA); mobile phase A: 95% water + 5% acetonitrile (containing 0.1% formic acid); mobile phase B: 47.5% acetonitrile + 47.5% isopropanol + 5% water (containing 0.1% formic acid); injection volume: 3 µL; column temperature: 40 °C.

The mass spectrometry signals of the samples were recorded using the mass scan range of *m*/*z* 70-1050 with positive and negative ion scanning modes. The spray voltages of the positive ion and the negative ion were kept at 3400 V and −3000 V, respectively. The gas sheath and the gas auxiliary heating were kept constant at 60 and 20 argon, respectively. The heater temperature of the ion source was set at 350 °C, and the collision energy was supplied as a series of 20-40-60 V. Raw data was manipulated with the help of the Progenesis QI (Waters Corporation, Milford, CT, USA) software to eliminate false positive peaks and internal standard peaks. The HMDB, Metlin, and Majorbio databases were used in order to identify metabolites.

PLS-DA was performed to characterize the metabolic separation between VMV-treated and PBS control groups in positive and negative ion modes. Model validation was conducted using cross-validation to calculate R^2^Y (model explanatory power) and Q^2^ (model predictive power), and permutation testing (200 cycles) assessed model overfitting; reliability was determined when the Q^2^ regression intercept was <0.05. The data resulting matrix was uploaded to the MajorBio Cloud platform (https://cloud.majorbio.com, Majorbio Cloud 2024, accessed on 27 March 2025) for analysis.

DEMs’ selection was based on the variable importance in projection (VIP) of the OPLS-DA model and the *p*-value of Student’s *t*-test (VIP > 1 and *p* < 0.05 were deemed significant). Database-based search queries (KEGG https://www.genome.jp/kegg/, Release 113.0, accessed on 12 March 2025) and Fisher’s exact tests in Python packages (https://docs.scipy.org/doc/scipy/, version 1.13.1, accessed on 12 March 2025) have been used to perform database-based enrichment and pathway analyses of such metabolites.

### 4.6. Integrated Network Analysis of the Transcriptome and Metabolome

Spearman correlation analysis has been performed to identify correlations between the key DEGs and metabolites. The correlation matrix that emerged out of this was depicted in R and Cytoscape (https://cytoscape.org/; Cytoscape software version 3.7.2) and this allowed seeing how the genes and the metabolites interacted in different ways such as heatmaps, hierarchical clusters, and correlation networks. Spearman correlation network analysis was conducted on those genes and metabolites that had important correlation coefficients (|ρ| ≥ 0.5, *p* < 0.01).

### 4.7. Real-Time Quantitative PCR (qPCR)

The expression levels of Icam, Cybb, Cyba, Ccl9, and Ccr1 mRNA were determined using the qRT-PCR approach. Total RNA was isolated as per the recommendation of the Eastep^®^ Super Total RNA extraction kit (Promega Corporation, Madison, WI, USA). Then, RNA samples were reverse transcribed with cDNA with the TransStart^®^ One-step gDNA Removal and cDNA Synthesis SuperMix (Transgen Biotech, Beijing, China) and amplified with specific primers with the use of the PerfectStart^®^ Green qPCR SuperMix (+Universal Passive Reference Dye) (Transgen Biotech). The synthesis of the RNA primers used in this research was conducted by Shanghai Sangon Biotechnology Co., Ltd. (Shanghai, China); their sequences are given in Supplementary Materials. The 2^−ΔΔCt^ method was used to determine the relative level of gene expression, which was normalized to reference gene expression of an internal reference gene.

### 4.8. Statistical Analysis

The analysis has been done in SPSS 27.0 through two-way repeated-measures ANOVA on body temperature. Where appropriate, Bonferroni post hoc multiple comparisons were conducted to identify statistically significant differences among VMV dose groups (40/60/80 μg). Graphs have been plotted using GraphPad Prism 10.1.2. The statistical significance was defined as the value of *p* < 0.05.

## Data Availability

The data presented in this study are available upon request from the corresponding author in summarised form, where possible, for reasons of privacy.

## Supplementary Materials

The following supporting information can be downloaded at: https://www.mdpi.com/article/10.3390/toxins18050198/s1, Figure S1: Differential Metabolites-Differentially Expressed Genes (DMs-DEGs) Correlation Chord diagram; Figure S2: Sample Test Results; Figure S3: Quality Inspection Report for RNA Samples; Figure S4: PLS-DA model validation by permutation test; Figure S5: Linear Regression Analysis of VMV Dosage and ΔTemp.
