# Supplementary material for: Transcriptome–Metabolome Integration Deciphers the Metabolic and Transcriptional Reprogramming in Mice Due to Vespa mandarinia Venom
Source: Toxins (Basel). 2026 Apr 23;18(5):198. doi: 10.3390/toxins18050198 (PMC13211639; doi:10.3390/toxins18050198)
Supplement: Supplementary file 1 [file toxins-18-00198-s001.zip › toxins-4217192-supplementary.pdf]

Supplementary Materials: Transcriptome–Metabolome Integration Deciphers the Metabolic and Transcriptional Reprogramming in Mice due to Vespa mandarinia Venom

Jisu Jin, Guangyuan Jiao, Xiaolei Huang, Yingying Sun, Chao Chen * and Hong Zhang *

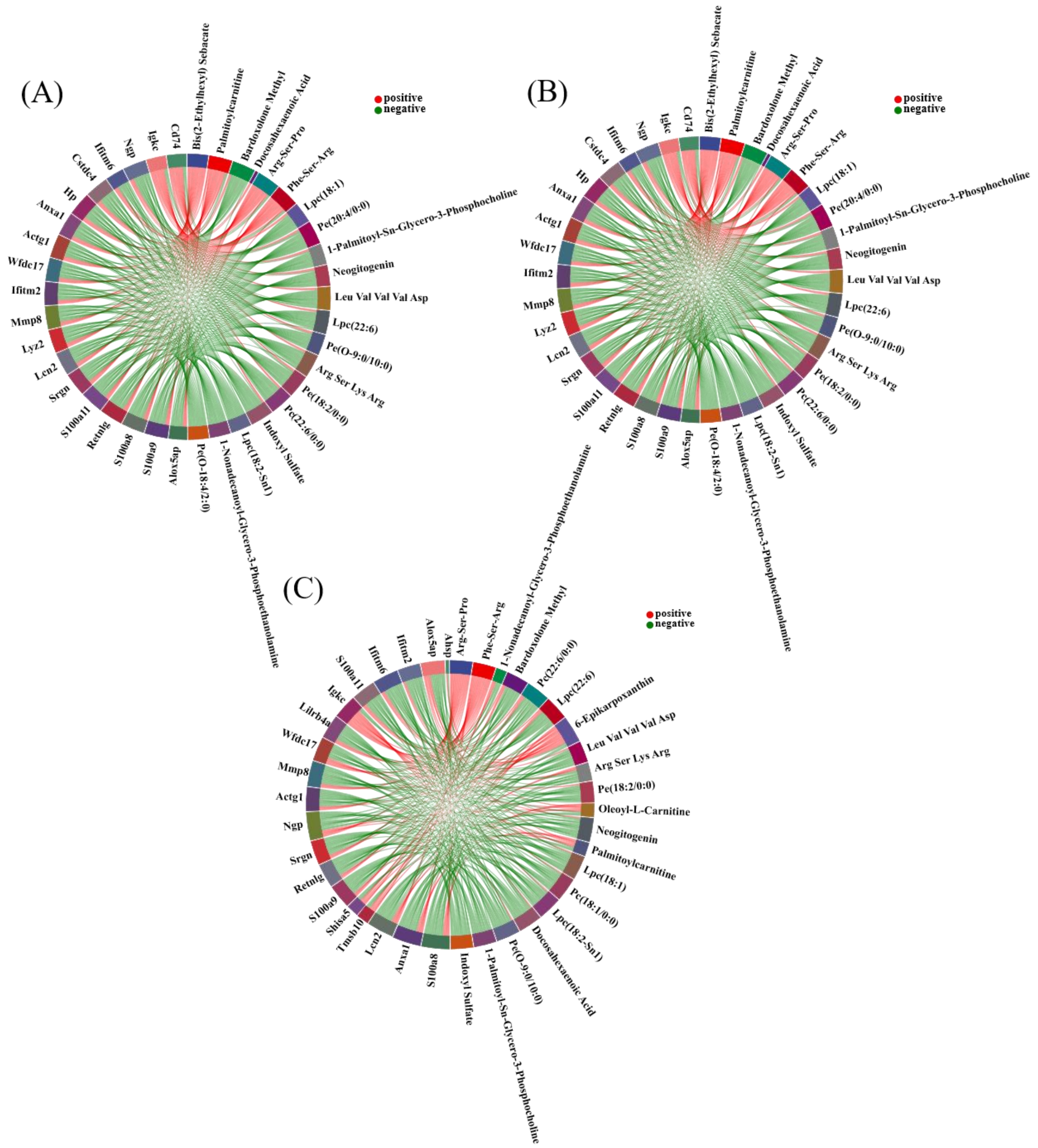

Supplementary Figure S1. Differential Metabolites-Differentially Expressed Genes (DMs-DEGs) Correlation Chord diagram. The diagram for (A) VMV 40 vs. PBS and (B) VMV 60 vs. PBS and (C) 80μgVMV vs. CK. The labels on the periphery of the diagram denote genes or metabolites, with metabolites positioned on the left and genes on the right. The connecting lines within the circles indicate correlations between genes and metabolites, where each chord

within a circle signifies a significant association between a metabolite and that particular gene. Red chords represent positive correlations, while green chords denote negative correlations.

Serial No.	MajorBio Sample ID	Sample Name	Sample Type	Concentration (ng/μL)	Total Amount (μg)	0D260/280	0D260/230	RQN (RNA Quality Number)	QC Result	Notes
1	EJA1800668	CK_1	Whole Blood	89.93	3.148	1.98	0.86	8.90	Pass	
2	EJA1800669	CK_2	Whole Blood	147.27	5.154	1.92	1.44	8.90	Pass	
3	EJA1800670	CK_3	Whole Blood	98.36	3.443	1.97	0.62	8.80	Pass	
4	EJA1800671	CK_4	Whole Blood	109.7	3.839	1.98	0.93	8.80	Pass	
5	EJA1800672	VMV40_1	Whole Blood	60.59	2.121	1.96	1.88	8.70	Pass	
6	EJA1800673	VMV40_2	Whole Blood	140.17	4.906	1.81	1.14	8.90	Pass	
7	EJA1800674	VMV40_3	Whole Blood	96.07	3.362	2.01	1.55	8.50	Pass	
8	EJA1800675	VMV40_4	Whole Blood	88.31	3.091	1.97	1.51	8.70	Pass	
9	EJA1800676	VMV60_1	Whole Blood	128.4	4.494	2.05	1.97	8.90	Pass	
10	EJA1800677	VMV60_2	Whole Blood	96.84	3.39	2.00	1.66	8.50	Pass	
11	EJA1800678	VMV60_3	Whole Blood	88.69	3.104	2.00	1.56	8.50	Pass	
12	EJA1800679	VMV60_4	Whole Blood	274.91	9.622	1.71	0.52	8.80	Pass	
13	EJA1800680	VMV80_1	Whole Blood	125.2	4.382	1.99	1.60	9.00	Pass	
14	EJA1800681	VMV80_2	Whole Blood	126.12	4.414	2.02	1.55	9.00	Pass	
15	EJA1800682	VMV80_3	Whole Blood	93.53	3.273	2.01	1.92	8.70	Pass	
16	EJA1800683	VMV80_4	Whole Blood	97.49	3.412	2.01	8.80	8.80	Pass	
Remarks:										

Supplementary Figure S2. Sample Test Results. RNA purity and concentration were determined using a NanoDrop 2000.

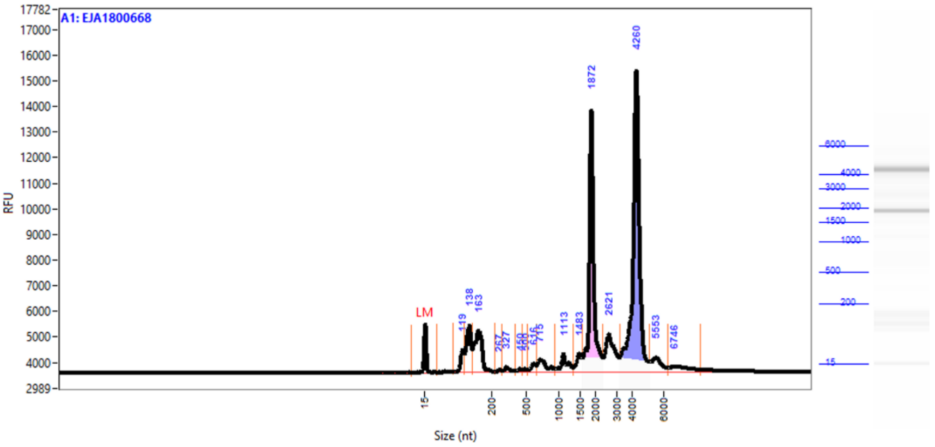
A1: EJA1800668
RFU
Size (nt)
LM
1872
4260
2621
5553
6746
1113
1483
138
163
119

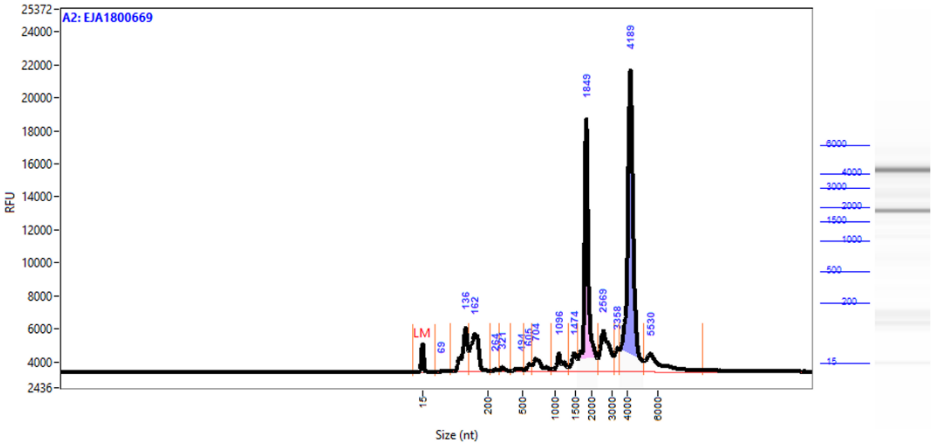
A2: EJA1800669
RFU
Size (nt)
LM
1849
4189
2569
3358
5530
1096
1474
136
162
69

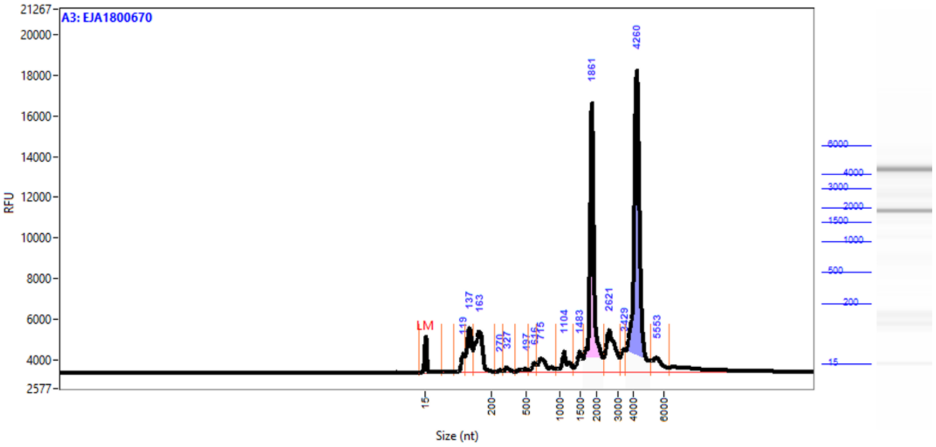
A3: EJA1800670
RFU
Size (nt)
LM
1861
4260
2621
5553
1104
1483
137
163
119

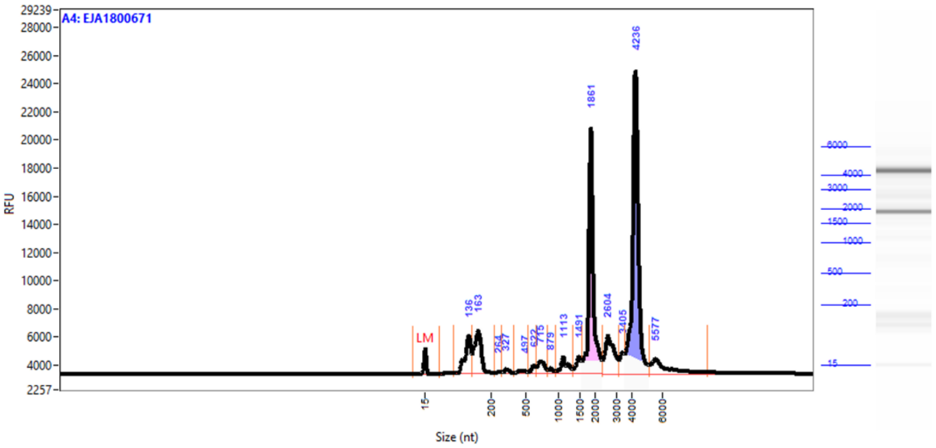
A4: EJA1800671
RFU
Size (nt)
LM
1861
4236
2604
3405
5577
1113
1491
136
163

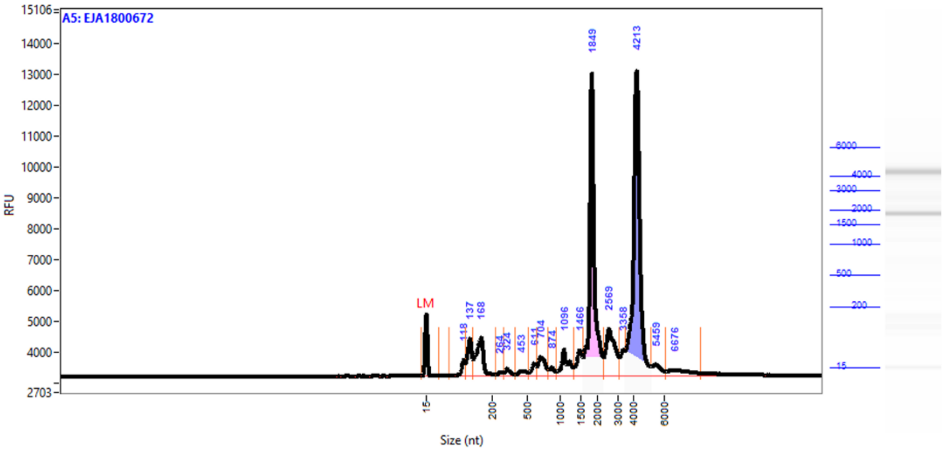
A5: EJA1800672
RFU
Size (nt)
LM
1849
4213
137
168
118
264
324
453
611
704
874
1096
1466
2569
3358
5459
6676
15
200
500
1000
1500
2000
3000
4000
6000

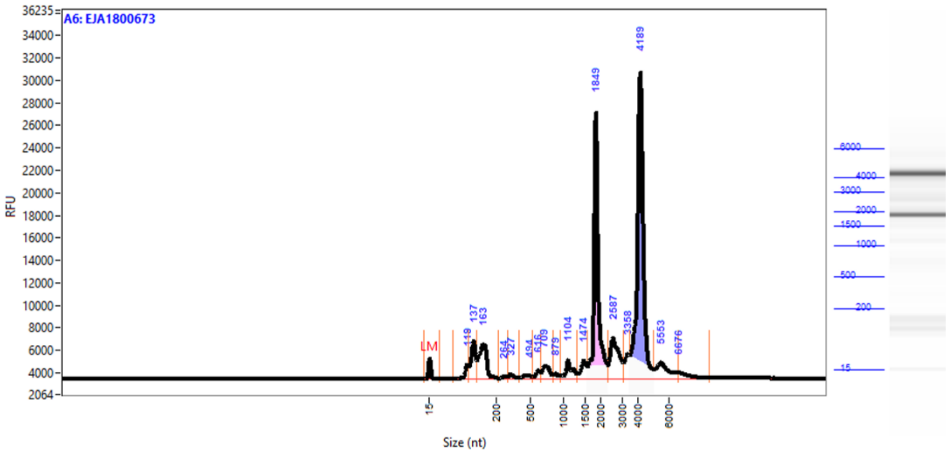
A6: EJA1800673
RFU
Size (nt)
LM
1849
4189
137
163
119
264
327
494
616
709
879
1104
1474
2587
3358
5553
6676
15
200
500
1000
1500
2000
3000
4000
6000

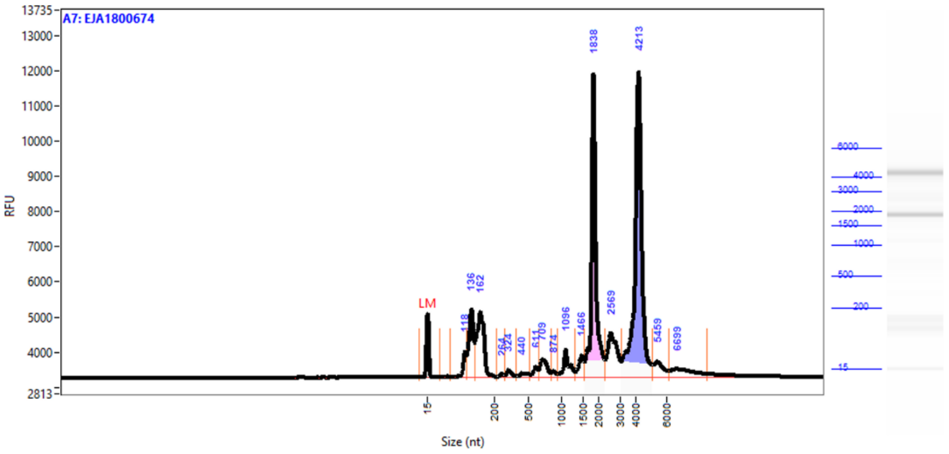
A7: EJA1800674
RFU
Size (nt)
LM
1838
4213
136
162
118
264
324
440
611
709
874
1096
1466
2569
5459
6699
15
200
500
1000
1500
2000
3000
4000
6000

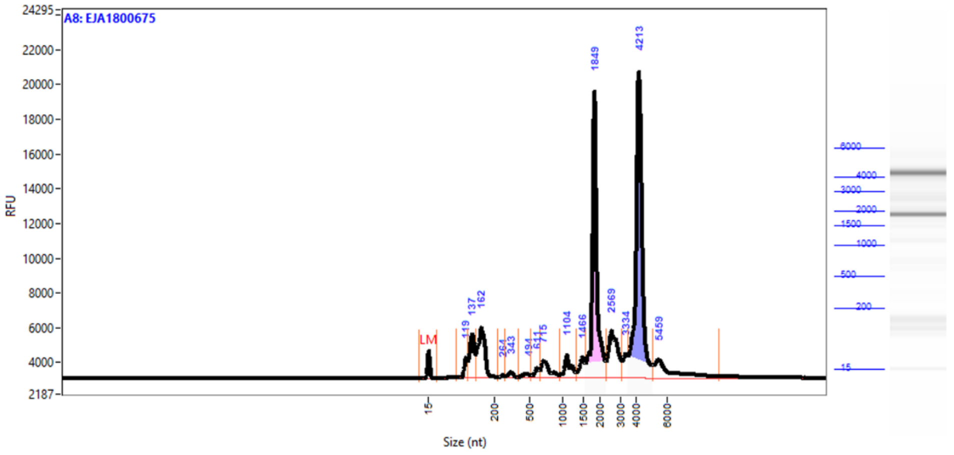
A8: EJA1800675
RFU
Size (nt)
LM
1849
4213
137
162
119
264
343
494
611
715
1104
1466
2569
3334
5459
15
200
500
1000
1500
2000
3000
4000
6000

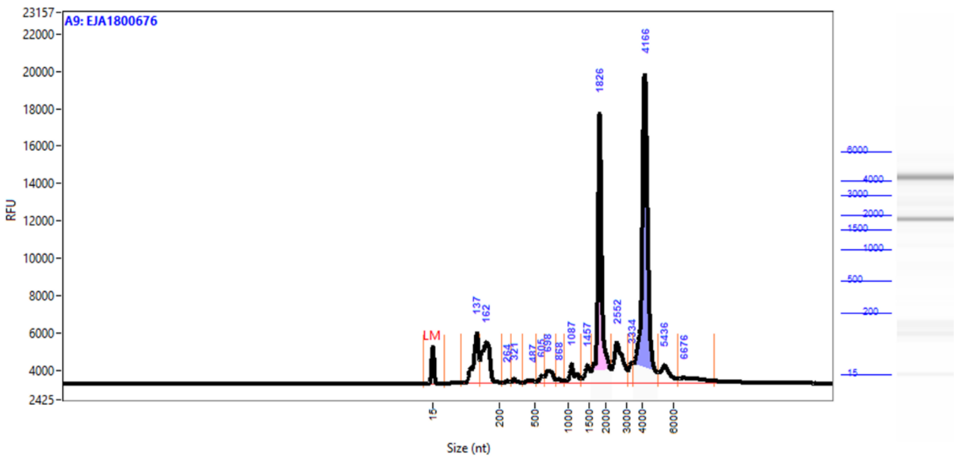
A9: EJA1800676
RFU
LM
137
162
1826
2552
4166
5436
6676
Size (nt)

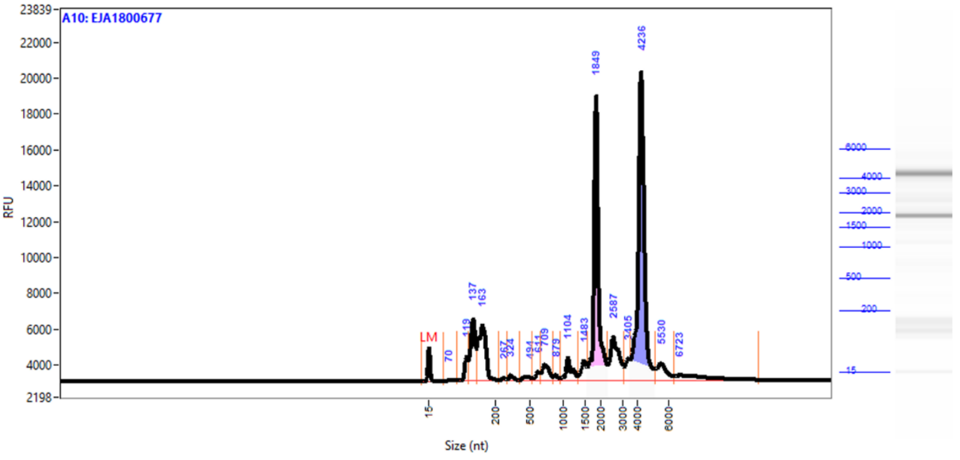
A10: EJA1800677
RFU
LM
137
163
1849
2587
4236
5530
6723
Size (nt)

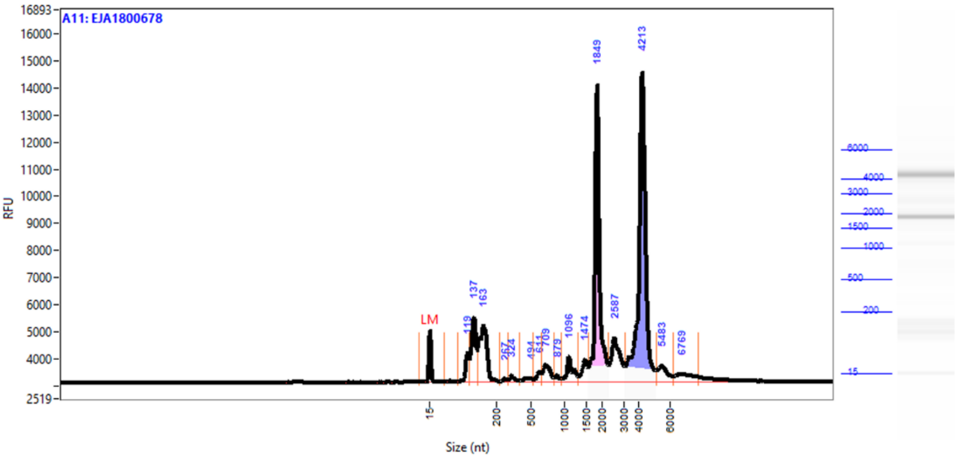
A11: EJA1800678
RFU
LM
137
163
1849
2587
4213
5483
6769
Size (nt)

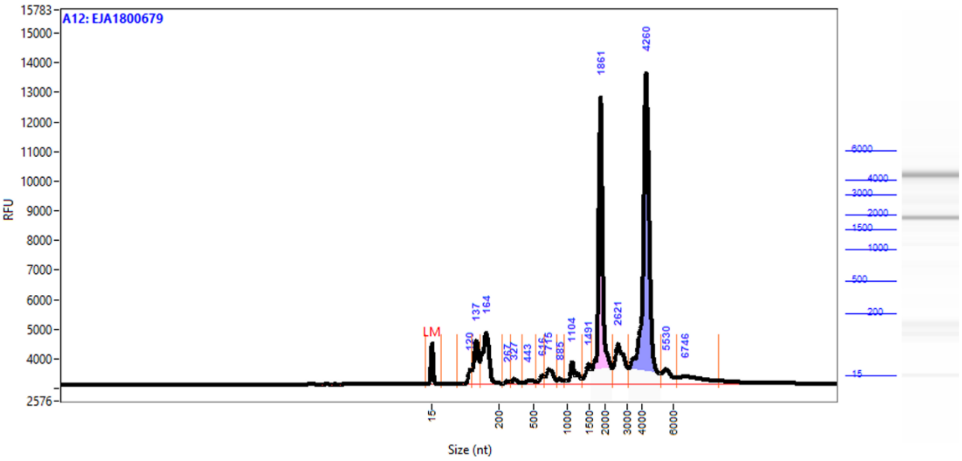
A12: EJA1800679
RFU
LM
137
164
1861
2621
4260
5530
6746
Size (nt)

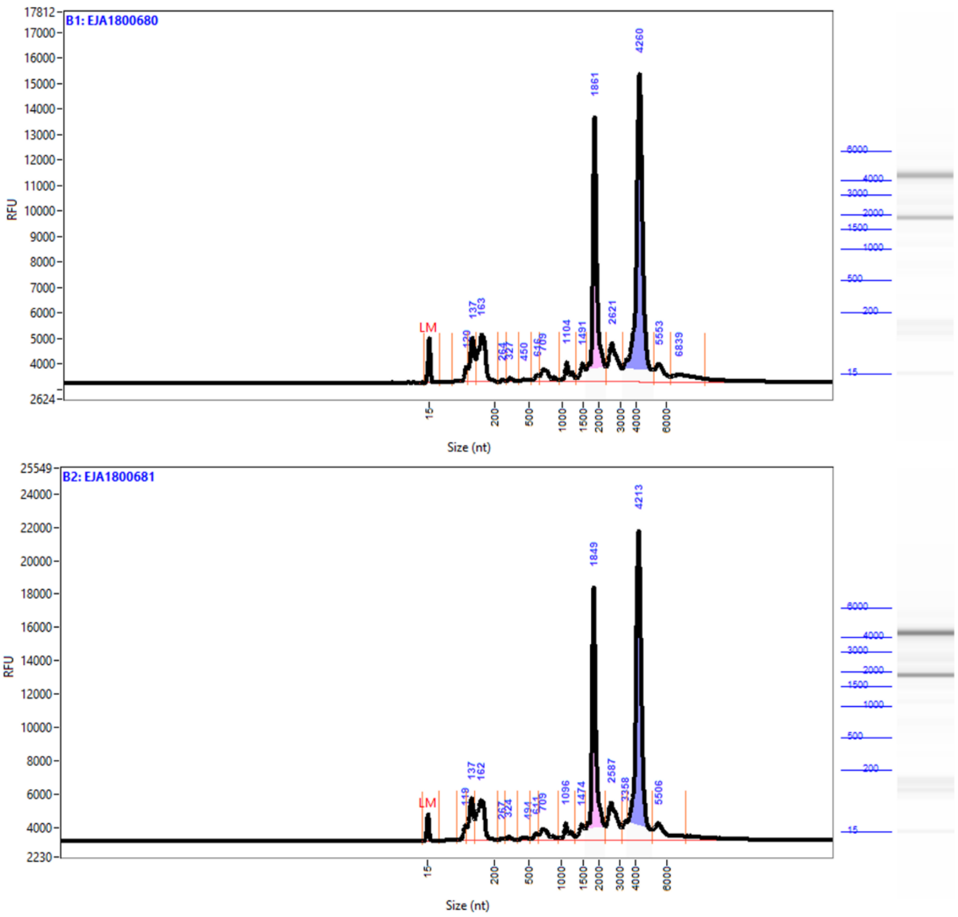

Supplementary Figure S3. Quality Inspection Report for RNA Samples. The concentration of each peak reflects the integrity of RNA in the sample; a more concentrated peak indicates a lower RNA degradation rate. The RQN value represents RNA integrity on a scale of 1–10, with higher values signifying better RNA integrity.

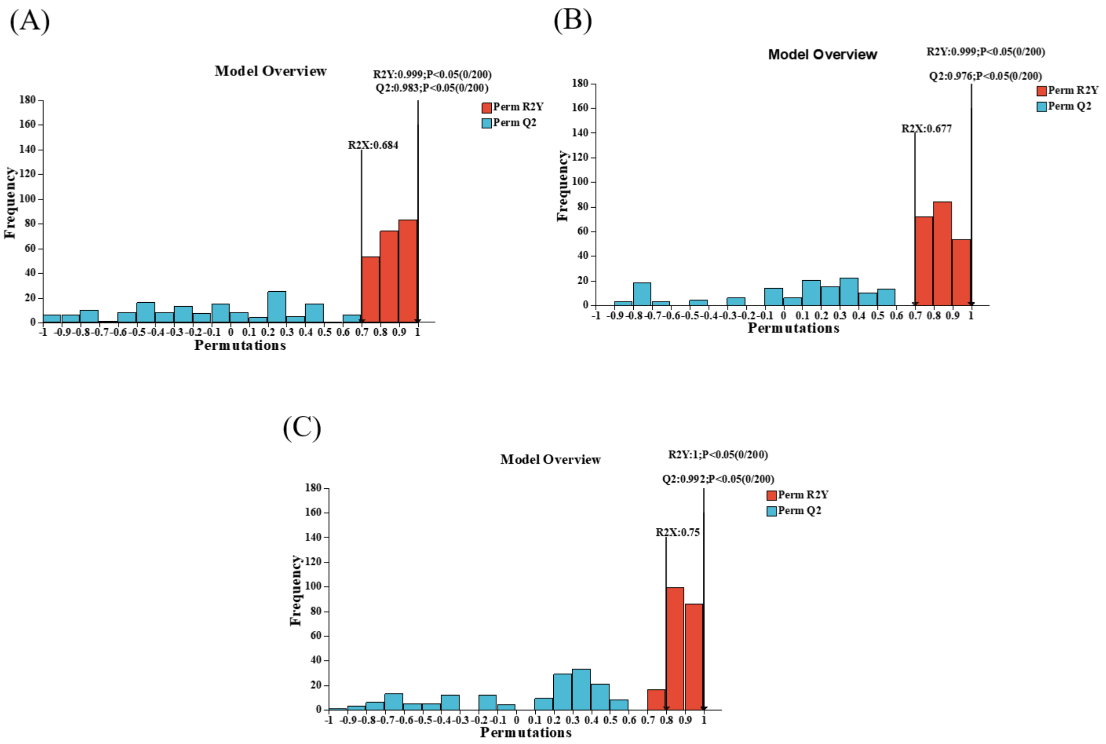

Supplementary Figure S4. PLS-DA model validation by permutation test. (A) VMV 40 vs. PBS, (B) VMV 60 vs. PBS, and (C) VMV 80 vs. PBS. The abscissa represents the accuracy of random models PLS-DA model validation by permutation test generated by permutation test, and the ordinate represents the number of corresponding random models. Red bars indicate the frequency of Q^2 values obtained from the permutation test, while blue bars represent the frequency of R^2Y values.

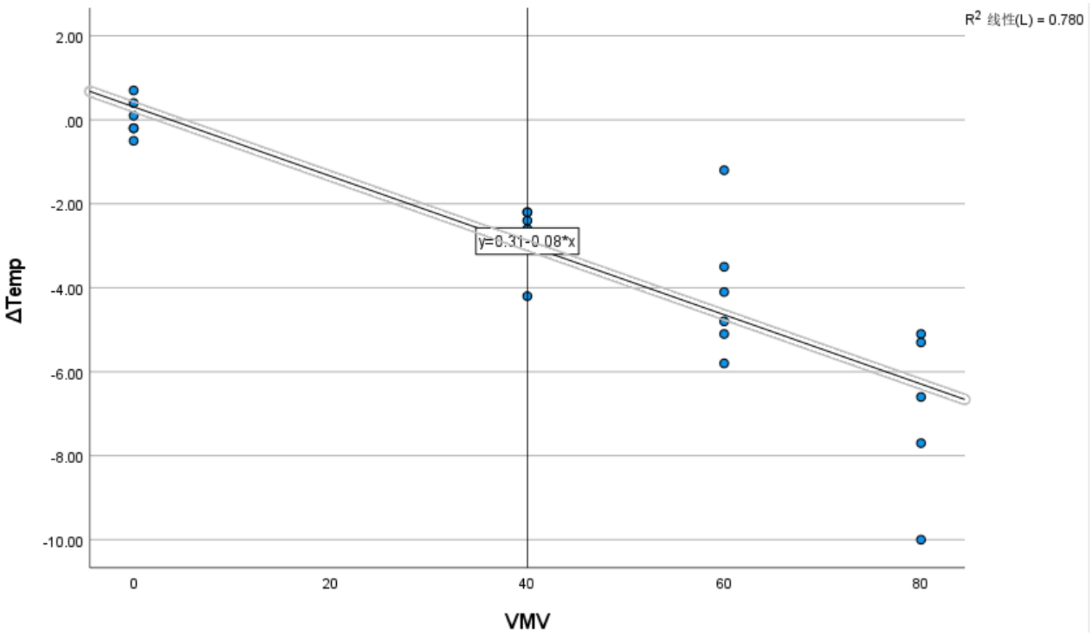

Supplementary Figure S5. Linear Regression Analysis of VMV Dosage and Δ Temp. Linear correlation analysis of VMV dosage with the measured index. A significant negative linear correlation was observed. Regression equation: Y = −0.08X + 0.3102, $R^2 = 0.780$, $p < 0.001$.
